# Synthesis of Poly(2-(methylsulfinyl)ethyl methacrylate) via Oxidation of Poly(2-(methylthio)ethyl methacrylate): Evaluation of the Sulfoxide Side Chain on Cryopreservation

**DOI:** 10.1021/acspolymersau.2c00028

**Published:** 2022-08-05

**Authors:** Toru Ishibe, Natalia Gonzalez-Martinez, Panagiotis G. Georgiou, Kathryn A. Murray, Matthew I. Gibson

**Affiliations:** † Department of Chemistry, University of Warwick, Gibbet Hill Road, CV4 7AL, Coventry, U.K.; ‡ Division of Biomedical Sciences, Warwick Medical School, University of Warwick, Gibbet Hill Road, CV4 7AL, Coventry, U.K.

**Keywords:** polymers, cryopreservation, RAFT, DMSO, macromolecular cryoprotectant

## Abstract

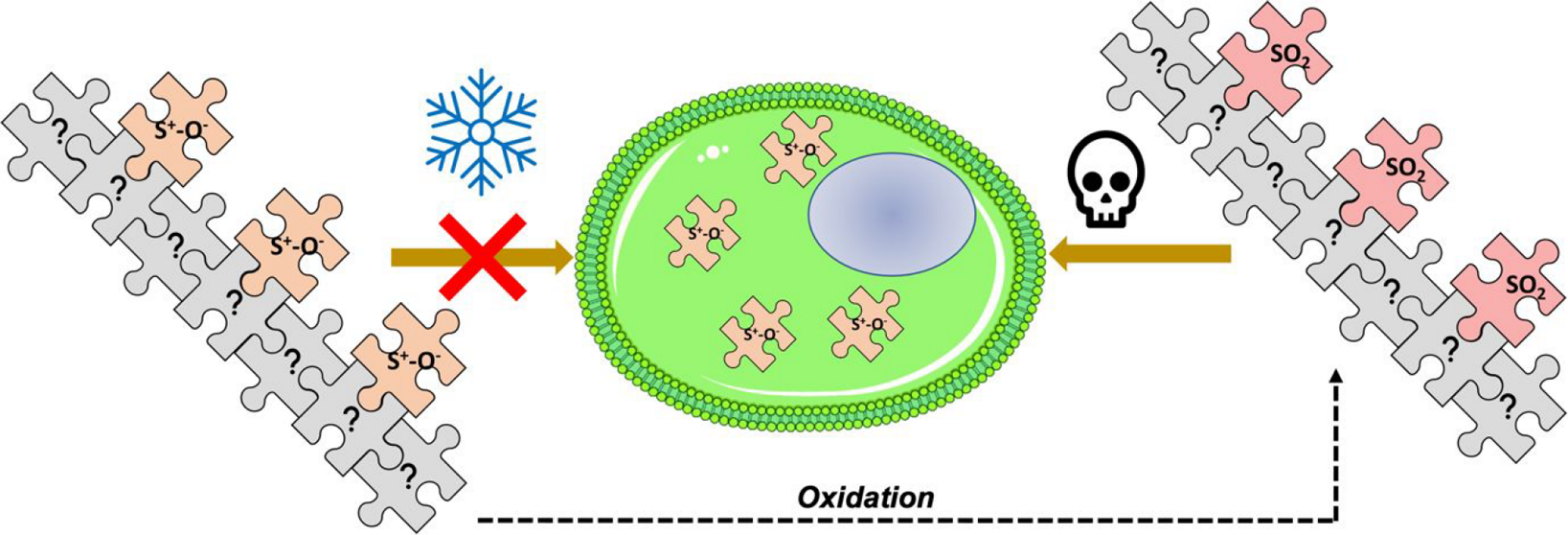

Conventional cryopreservation solutions rely on the addition of organic solvents such as DMSO or glycerol, but these do not give full recovery for all cell types, and innovative cryoprotectants may address damage pathways which these solvents do not protect against. Macromolecular cryoprotectants are emerging, but there is a need to understand their structure–property relationships and mechanisms of action. Here we synthesized and investigated the cryoprotective behavior of sulfoxide (i.e., “DMSO-like”) side-chain polymers, which have been reported to be cryoprotective using poly(ethylene glycol)-based polymers. We also wanted to determine if the polarized sulfoxide bond (S^+^O^–^ character) introduces cryoprotective effects, as this has been seen for mixed-charge cryoprotective polyampholytes, whose mechanism of action is not yet understood. Poly(2-(methylsulfinyl)ethyl methacrylate) was synthesized by RAFT polymerization of 2-(methylthio)ethyl methacrylate and subsequent oxidation to sulfoxide. A corresponding *N*-oxide polymer was also prepared and characterized: (poly(2-(dimethylamineoxide)ethyl methacrylate). Ice recrystallization inhibition assays and differential scanning calorimetry analysis show that the sulfoxide side chains do not modulate the frozen components during cryopreservation. In cytotoxicity assays, it was found that long-term (24 h) exposure of the polymers was not tolerated by cells, but shorter (30 min) incubation times, which are relevant for cryopreservation, were tolerated. It was also observed that overoxidation to the sulfone significantly increased the cytotoxicity, and hence, these materials require a precision oxidation step to be deployed. In suspension cell cryopreservation investigations, the polysulfoxides did not increase cell recovery 24 h post-thaw. These results show that unlike hydrophilic backboned polysulfides, which can aid cryopreservation, the installation of the sulfoxide group onto a polymer does not necessarily bring cryoprotective properties, highlighting the challenges of developing and discovering macromolecular cryoprotectants.

## Introduction

Cryopreservation dramatically slows kinetics and halts cellular metabolism, allowing stable long-term storage of cellular material.^1^ Cryopreservation avoids the epigenetic changes and phenotype drift that occur when cells are extensively cultured, which improves reliability and reproducibility in multiple biomedical research areas.^2^ Recent advances in the development of cell-based therapies, where patient or donor cells are genetically modified *ex vivo* and infused into the patient, have highlighted the importance of a robust cryopreservation workflow.^3^ Cryopreservation allows long-term storage of cellular products before and after modification, which enables product transport from the manufacturing site to the clinic, extends product life, and provides flexibility in patient treatment.^4^ The most widely used cryoprotective agent is dimethyl sulfoxide (DMSO), which is used in both “slow freezing” applications (5–10 wt %), where ice is allowed to form, and in vitrification (>20 wt %), where a glassy, ice-free, state is achieved by using high freezing rates.^5,6^ DMSO can permeate cells to reduce osmotic stress during freezing by replacing intracellular water. DMSO is highly successful but does have side effects^7−9^ and is cytotoxic,^10^ and hence, there is a desire to replace it or reduce the quantity needed. Additionally, not all cell types show high recovery with DMSO-mediated cryopreservation, for example, the RAW 264.7 cell line and isolated leukocytes are especially sensitive to DMSO.^11^

Polymeric additives in cryopreservation are well-known, including poly(vinylpyrrolidone)^12^ and hydroxyethyl starch,^13,14^ but new macromolecular cryoprotectants with advanced function are emerging.^15^ Ice-binding proteins,^16^ which can modulate the formation and growth of ice, have attracted significant attention since it was demonstrated that they can reduce ice recrystallization during thawing of cells,^17^ although ice recrystallization inhibition alone fails to explain the cryoprotective outcomes.^18,19^ Synthetic mimics of ice-binding proteins have attracted attention because of their scalability and the ease of tuning their structure.^20−22^ For example, poly(vinyl alcohol),^23−25^ self-assembling peptides,^26^ and oligoproline^27,28^ have all shown some benefit in cryopreservation. Small-molecule ice growth inhibitors from Ben and co-workers have been used to cryopreserve red blood cells because of their IRI activity.^29,30^ Matsumura and co-workers introduced polyampholytes (polymers with mixed cationic/anionic groups), which are very potent cryoprotectants^31^ in both slow freezing^32^ and vitrification^33^ applications. Furthermore, the polysaccharide fucopol has been applied to cryopreserve various cell lines.^34^

An interesting class of polymer biomaterials are those containing sulfides/sulfoxides. Hubbell and co-workers showed that main-chain sulfide polymers can oxidize to the more hydrophilic sulfoxide as a disassembly trigger^35^ and respond to oxidative microenvironments to release payloads.^36^ Redox-responsive polymers have been widely explored for drug delivery.^37^ Lynd and co-workers have reported the synthesis of poly(methyl glycidyl sulfoxide), essentially a poly(ethylene glycol) backbone with sulfoxide side chains, and applied it to cryopreservation.^38^ The hypothesis was that a DMSO-like side chain could provide some of the benefits of DMSO, despite the fact that hydrophilic polymers are unlikely to be cell-penetrative, which is key to DMSO’s function. This material showed promisingly high immediate post-thaw viabilities when 3T3 cells were cryopreserved (DMSO-free), but it has been validated that immediate post-thaw cell recovery is a poor predictor of cryopreservation outcomes, as many cells cannot be cultured.^39^ Analysis 24 h post-thaw revealed the polymer was 50% as effective as DMSO, as opposed to being more potent in immediate post-thaw assays. The result is still significant as any post-thaw recovery in DMSO-free conditions is hard to achieve and hence shows potential. However, there remain few studies on whether sulfoxide side chains can play a specific role in macromolecular cryoprotectants. Such a study would also aid our understanding of cryoprotective polyampholytes^15^ that have segregated anionic/cationic groups, whereas the sulfoxide has a highly polarized sulfur-oxygen bonding, which can be considered to have dipolar character and hence charge on the S/O atoms.

Considering the above, here we investigated poly(2-(methylsulfinyl)ethyl methacrylate) (PMSEM) in the context of its cryopreservation potential. PMSEM was synthesized by controlled oxidation of poly(2-(methylthio)ethyl methacrylate). Overoxidation (to the sulfone) led to significant cytotoxicity against A549 cells, highlighting the importance of this synthetic step. A control polymer with an *N*-oxide side chain (PDMAOEM) was synthesized in order to further test the impact of a dipolar side chain on cryoprotective ability. PMSEM was evaluated for ice recrystallization inhibition activity and total ice fraction formation, revealing that the DMSO-like side chain had essentially no impact on ice formation and growth. Suspension cell cryopreservation assays revealed that the DMSO-like polymers failed to significantly improve cell recovery in a low DMSO (2.5 wt %) model cryopreservation study, although a rather large range of recovery values was obtained. This suggests that simply including sulfoxides into synthetic polymers does not bring the benefits of the small molecule DMSO and that the polarized side chains of sulfoxide and *N*-oxides do not bring the benefits of the mixed-charge side chains found in cryoprotective polyampholytes.

## Results and Discussion

**Figure 1:**
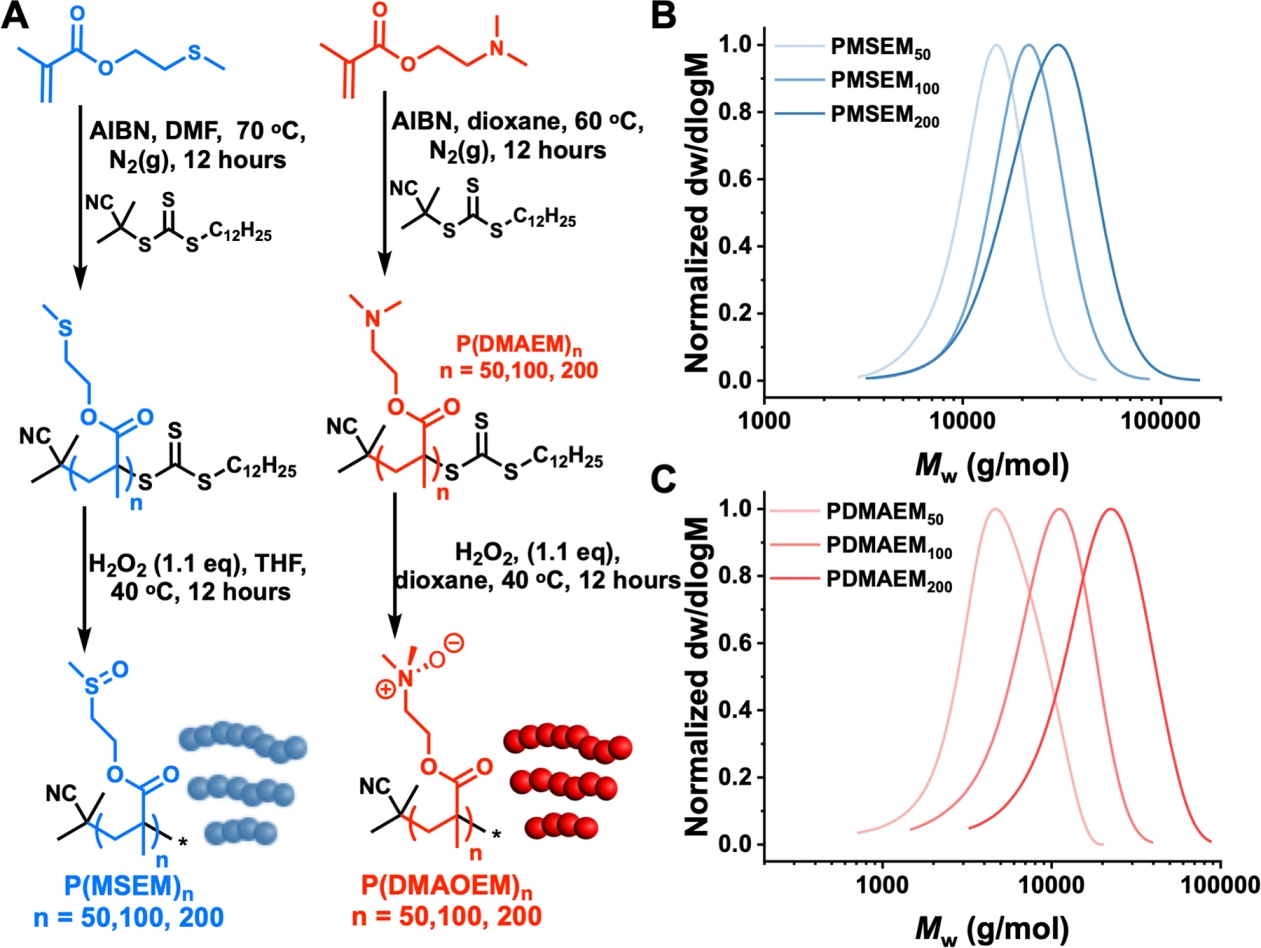
Synthesis of sulfoxide and control amine-oxide polymers. (A) Polymer synthesis and oxidation. Molecular weight distributions from size exclusion chromatography of (B) PMSEM and (C) PDMAEM.

As our aim was to investigate if sulfoxide side-chain polymers could have cryoprotective benefit, a synthetic strategy was devised from a thio-ether precursor. 2-(Methylthio)ethyl methacrylate was polymerized^40^*via* via thermally initiated RAFT polymerization using 2-cyano-2-propyl dodecyl trithiocarbonate and 2,2′-azobis(2-methylpropionitrile) (AIBN), Figure 1A. As a control, PDMAEM (poly(2-(dimethylamino)ethyl methacrylate)) was also synthesized, as the tertiary amine can also be oxidized, providing a useful comparison to the sulfoxide polymer. A sulfoxide can be considered to be charge-separated S^+^O^–^, and hence, an amine-oxide is a suitable control polymer to replicate this charge separation. This also allows us to make a comparison with polyampholytes, which are mixed charge polymers that are very potent cryoprotectants, and separate any unique features of the sulfoxide from the charge separation. The polymers were characterized by size exclusion chromatography (Figure 1B,C) showing monomodal distributions and narrow dispersities. Molecular weight data is shown in Table 1. The polymers were subsequently oxidized using a small excess (1.1 mol equiv) of H_2_O_2_^41^ with successful oxidation confirmed by ^1^H NMR and IR analysis, resulting in the formation of poly(2-(methylsulfinyl)ethyl methacrylate) (PMSEM) and (poly(2-(dimethylamineoxide)ethyl methacrylate)) (PDMAOEM) derivatives (Figures S3, S4). Note that oxidation is discussed further in the sections below, including overoxidation of PMSEM. SEC analysis in THF (+ 2% v/v NEt_3_) was performed on preoxidized PDMAEM derivatives. Control experiments using liposomes were undertaken as simplistic cell models. Figure S1 shows DOPC liposomes with and without addition of PMSEM. This shows that the polymers did not disrupt nor aggregate the liposomes, which was essential to allow further freeze/thaw testing, Figure S2.

**Table 1:** Polymers Synthesized by RAFT Polymerization

name	[M]:[CTA]	conversion (%)^a^	*M*_n,theo_ (g mol^–1^)^b^	*M*_n,SEC RI)_ (g mol^–1^)^c^	*Đ*^c^
polyMSEM_50_	50	96	8500	12000	1.19
polyMSEM_100_	100	96	17000	18000	1.21
polyMSEM_200_	200	99	35000	23000	1.33
polyDMAEM_50_	50	84	8200	4000	1.38
polyDMAEM_100_	100	65	16100	7700	1.40
polyDMAEM_200_	200	74	31800	16500	1.42

a Determined by ^1^H NMR using DMF as internal standard.

b Determined from the feed ratio of the monomer to chain-transfer agent assuming 100% conversion.

c *M*_n_ and *Đ*_M_ values for PMSEM polymers calculated against poly(methylmethacrylate) standards using 5 mM NH_4_BF_4_ in DMF as an eluent. *M*_n_ and *Đ*_M_ values for PDMAEM polymers calculated against poly(styrene) standards using THF + 2% v/v NEt_3_ as the eluent.

**Figure 2:**
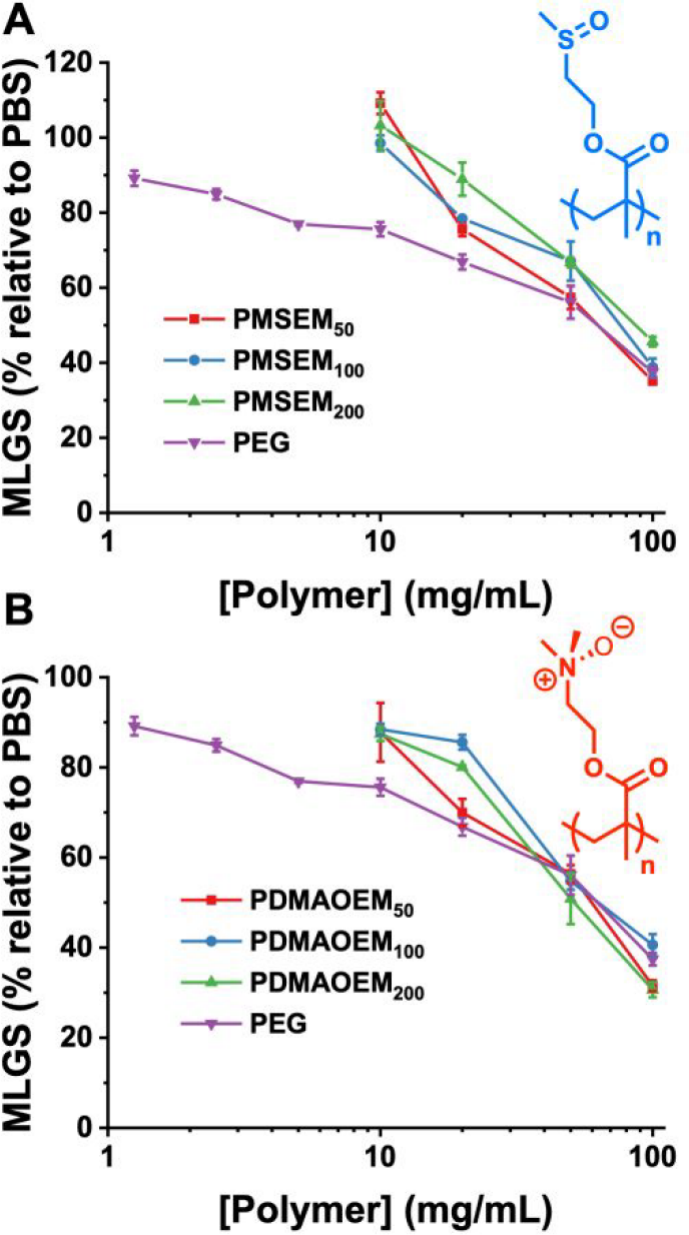
Ice recrystallization inhibition (IRI) activity. (A) Poly(2-(methylsulfinyl)ethyl methacrylate) (PMSEM). (B) (Poly(2-(dimethylamineoxide)ethyl methacrylate)) PDMAOEM. All data is reported relative to a PBS negative control, *n* = 3.

To evaluate the impact of the PMSEM and PDMAOEM on ice growth, a “splat” assay was used.^42,43^ A 10 μL droplet of the polymer in PBS (phosphate-buffered saline, [NaCl] = 0.137M) was dropped onto a cooled surface to generate a polynucleated wafer of ice. This was annealed at −8 °C for 30 min, and the mean largest grain size (MLGS, note this is subtly different than using mean grain size^44^) was determined relative to crystals obtained in PBS alone. Poly(ethylene glycol) (PEG) was used as a negative control for an inhibitor, which is crucial: any polymer at sufficiently high concentration will inhibit ice growth, and false positives can be reported if negative controls of an additional polymer (not just versus the PBS blank) are not used.^44^ As can be seen in Figure 2A,B, both PMSEM and PDMAOEM have no IRI activity relative to PEG but could be interpreted as having IRI at the highest concentrations if only being compared with PBS solutions. Neither of the polymers have obvious ice-binding sites, and the polymers are not facially amphiphilic, which are two structural motifs associated with IRI activity.^45^ It should be noted, however, that IRI activity alone is not a predictor of cryoprotective effect,^18^ and many polymers with no or little IRI are potent cryoprotectants.^15,34^ For instance, polyampholytes, with mixed cationic/anionic groups on the backbone, are potent cryoprotectants but only weak/moderate IRIs.^46,47^ Both polymers here (if the sulfoxide is drawn as a single-bonded S^+^O^–^) have mixed charges; this suggests that the distribution of charges in polyampholytes is crucial to their function, which is not replicated by the present materials.

The addition of conventional cryoprotectants, such as DMSO, has many functions, including reducing the total volume of ice formed by promoting a vitrified or nonfrozen state. Lynd and co-workers showed that a sulfoxide PEG derivative reduced the total water fraction frozen and impacted the *T*_g_, which they linked to cryopreservation potential.^38^ To explore the present polymers further, we used differential scanning calorimetry to evaluate the total ice formed in aqueous solutions frozen (Figures S5, S6). The total integral (proportional to ice volume) during both freezing and thawing cycles was measured and is shown below in Table 2. In all cases, there was a decrease in ice fraction compared with water alone, as would be expected upon addition of a water-soluble polymer. Compared with the PEG control, there were no significant changes, with all polymers performing approximately equally in reducing the total ice fraction formed. This data also supports the observations of Lynd that a PEG-backbone polymer with side-chain sulfoxides reduced the ice fraction formed and that the PEG component was essential.^38^ This does not discount that reducing the ice fraction can be a positive, but rather, it shows that the present polymers do not have a significant effect compared with other materials.

**Table 2:** Ice Formed upon Freezing from Differential Scanning Calorimetry^a^

sample	freezing area (J/g)	thawing area (J/g)
water	279.6	316.0
PMSEM_50_	226.0	244.6
PMSEM_100_	184.8	195.0
PMSEM_200_	205.6	210.8
PDMAOEM_50_	181.8	186.0
PDMAOEM_100_	187.2	189.0
PDMAOEM_200_	208.1	209.8
PEG	177.1	191.3

a Freezing to −40 °C at 0.5 °C min^–1^ and heating at 0.5 °C min^–1^.

**Figure 3:**
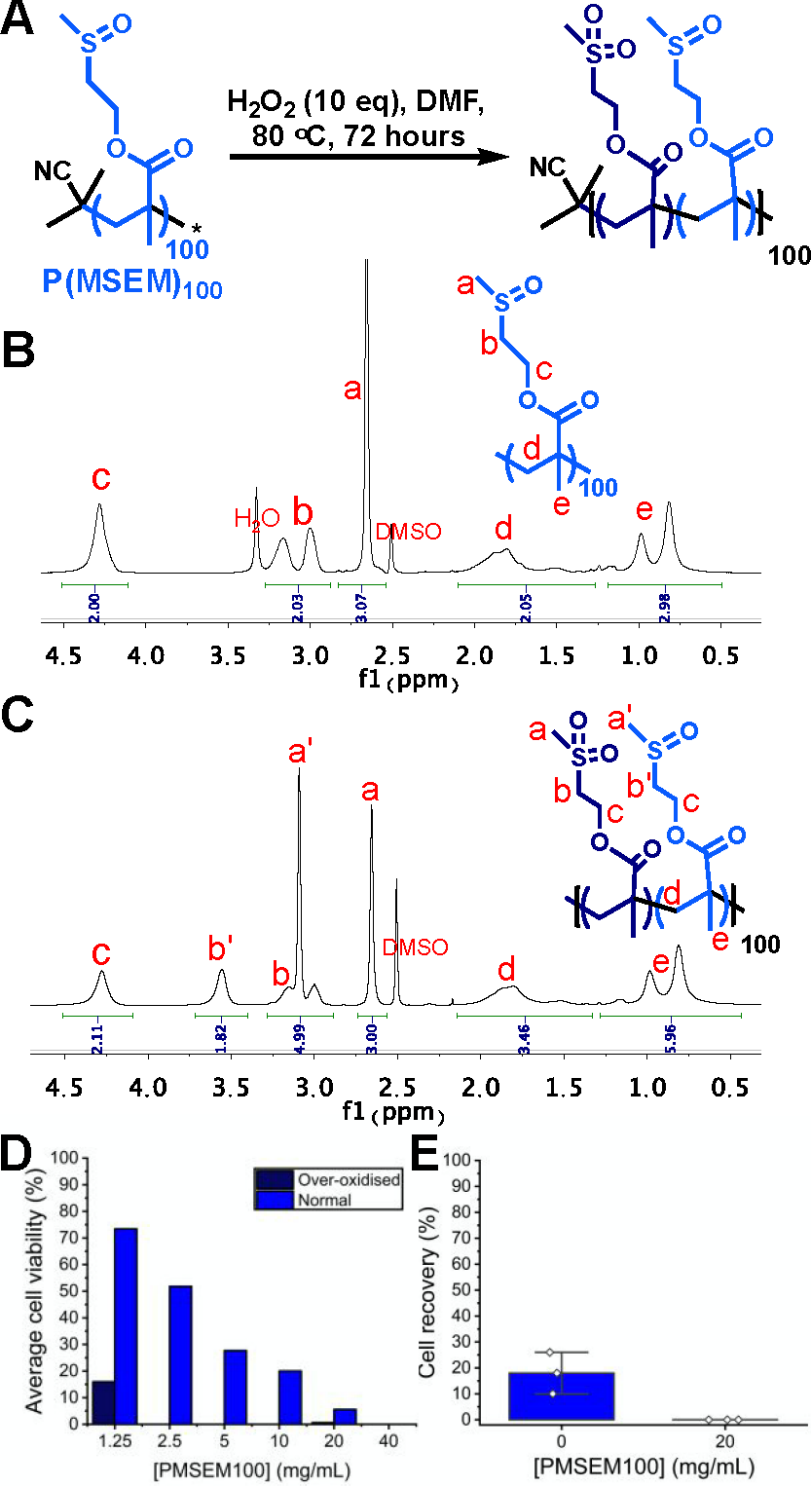
Impact of overoxidation on cytotoxicity. (A) Synthesis of sulfone/sulfoxide copolymers. (B) ^1^H NMR of sulfoxide polymer. (C) ^1^H NMR of sulfone/sulfoxide copolymers indicating characteristic peaks attributed to the sulfones. (D) Cytotoxicity of polymers against A549 cells after 24 h, determined by a resazurin reduction assay. (E) Post-thaw cell recovery after cryopreservation of A549 cells with overoxidized polymer and 2.5 wt % DMSO, determined using the trypan blue exclusion test.

Guided by the above, we then moved onto evaluating the cytotoxicity of PMSEM and its effect on cryopreservation. During oxidation from thio-ether to sulfoxide, it is possible to overoxidize to a sulfone, and hence, ensuring a single species (sulfoxide or sulfone) is crucial to evaluate the impact of this on any biological outcomes. Figure 3A shows the synthesis of PMSEM and deliberate overoxidation by addition of excess H_2_O_2_. Figure 3B shows a homogeneous sulfoxide (desired) polymer with a single methyl peak at ∼2.7 ppm. In the overoxidized case (Figure 3C), a second methyl peak can be seen at ∼3.1 ppm, showing that both sulfoxide and sulfone are present. Corresponding shifts in the methylene adjacent to the sulfur atom can also be seen. Figure 3D shows the analysis of cytotoxicity of PMSEM_100_ and its overoxidized (sulfone) equivalent against A549 (adenocarcinomic human alveolar basal epithelial) cells after 24 h of incubation. Note that 24 h is a far longer exposure than that typically used in cryopreservation, but this period allows rigorous comparison. From this initial testing, it was clear that overoxidation led to far more toxic polymers, with any concentration above 1.25 mg mL^–1^ leading to no viable cells. Overoxidation can also lead to cleavage of main-chain polysulfides, and hence, additional breakdown products cannot be ruled out here either.^48^ Initial cell cryopreservation studies using the overoxidized polymers also confirmed this cytotoxicity, with particularly low (almost zero) post-thaw recoveries being observed (Figure 3E). These results are crucial as they highlight how, when preparing sulfoxide polymers, overoxidation has a significant negative impact on cell recovery.

**Figure 4:**
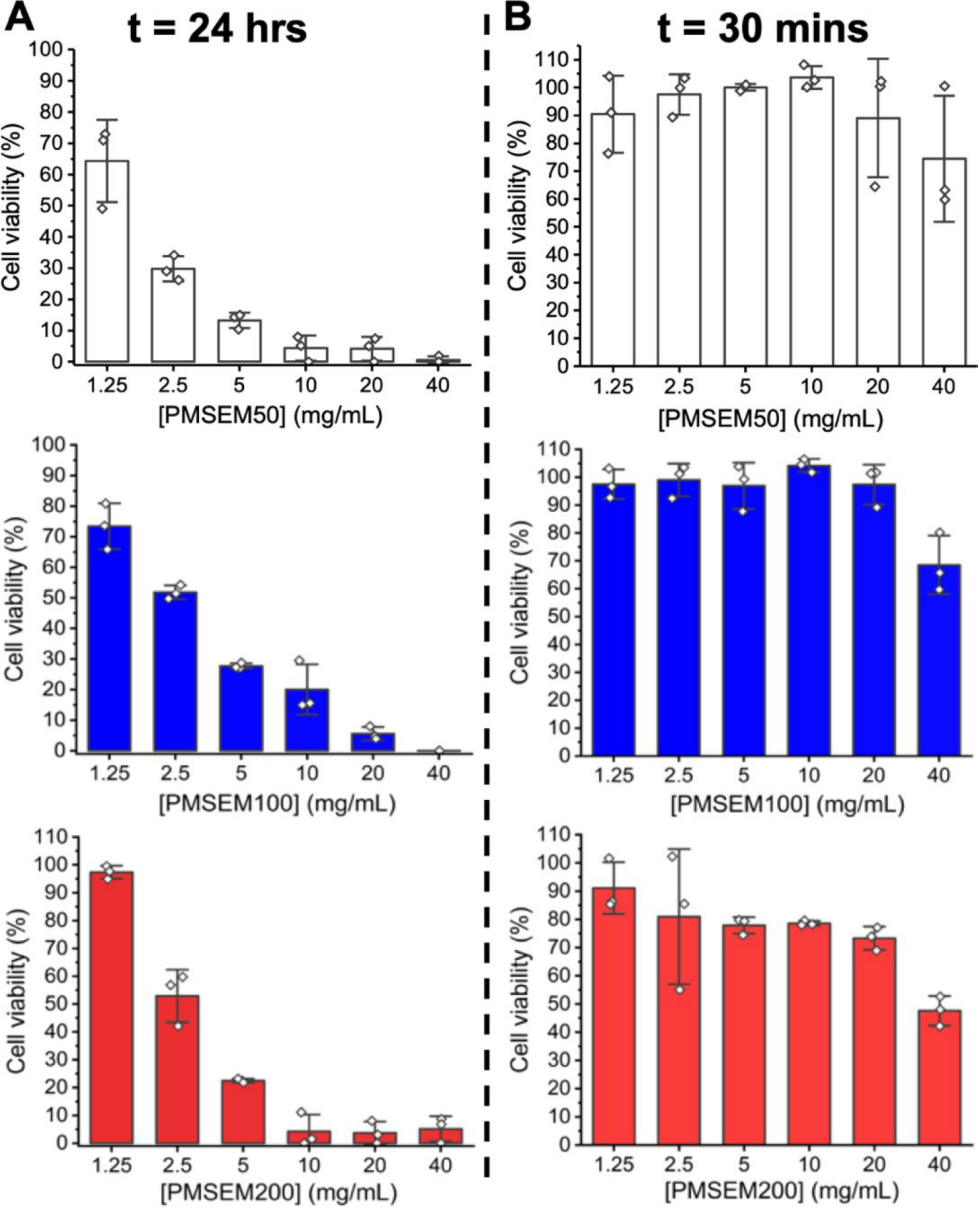
Cytotoxicity of sulfoxide polymers (from Table 2) as a function of molecular weight and exposure time on A549 cells. (A) 24 h exposure; (B) 30 min exposure. Viability was determined by a resazurin reduction assay and reported relative to a negative (no polymer) control. Results expressed as mean ± SD.

Single-oxidation polyMSEM polymers (confirmed by ^1^H NMR) were prepared for further testing, Table 3. Cytotoxicity was measured against A549 cells after 24 h incubation (to exaggerate any effects) and also 30 min, which is a realistic exposure time for the cells before freezing during cryopreservation (Figure 4). After 30 min of exposure, there was no trend between molecular weight and cytotoxicity, with all polymers being tolerated up to 20 mg mL^–1^, but at 40 mg mL^–1^, there was a decrease in cell viability. It should be noted that this response range is acceptable for a cryopreservation scenario, as seen for polyampholytes, which are very potent cryopreservation enhancers but cannot be left in extended culture.^49^ After 24 h of exposure, there was a clear dose-dependent response to the polymers with concentrations above 1.25 mg mL^1^ leading to decreased cell viability. Li *et al*. have previously prepared an acrylate equivalent^50^ (poly(2-(methylsulfinyl)ethyl acrylate) which shows low cytotoxicity, but it was only tested up to 3 mg mL^–1^. Main chain sulfoxides (by oxidation of poly(propylene sulfide)^48^ showed a similar decrease in cell viability to what is seen here when exposed to concentrations as high as 40 mg mL^–1^.

**Table 3:** PMSEM Homopolymers Examined for Cryopreservation

name	[M]:[CTA]	conversion (%)^a^	*M*_n,theo_ (g mol^–1^)^b^	*M*_n,SEC RI)_ (g mol^–1^)^c^	*Đ*_M_^c^
polyMSEM_50_	50	99	8500	12800	1.14
polyMSEM_100_	100	96	17000	17500	1.18
polyMSEM_200_	200	99	35000	23800	1.24

a Determined by ^1^H NMR using DMF as internal standard.

b Determined from the feed ratio of the monomer to chain-transfer agent assuming 100% conversion.

c *M*_n_ and *Đ*_M_ values calculated against poly(methylmethacrylate) standards using 5 mM NH_4_BF_4_ in DMF as an eluent.

**Figure 5:**
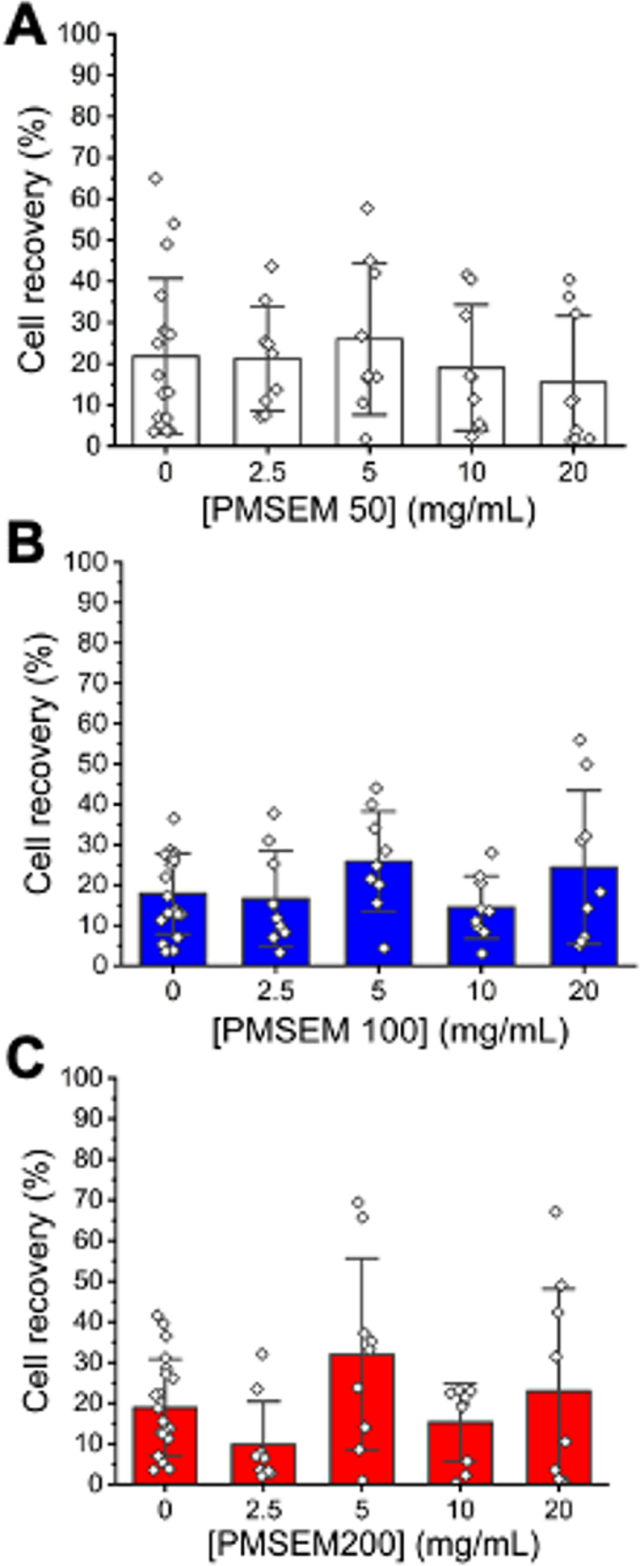
Cryopreservation of A549 cells (in suspension with 2.5 wt % DMSO) as a function of sulfoxide polymer molecular weight (from Table 3). Cell recovery was determined 24 h post-thaw using the trypan blue exclusion test. Results are expressed as mean ± SD. Plots show three biological repeats with three technical replicates each.

With the above data in hand, cryopreservation could be undertaken. Lynd *et al*. reported that sulfoxide side chain PEGs can be deployed in DMSO-free cryopreservation.^38^ In this previous report, the polymers led to less than 50% of the cell recovery compared with DMSO. This observation is common with polymer cryoprotectants, where if short (immediate post-thaw) viabilities are measured then high recoveries can be reported, but upon culture (24 h post-thaw), few cells propagate which are healthy. We recently demonstrated that a 24 h post-thaw culture is essential to remove false-positives with macromolecular cryoprotectants.^39^Figure S8 compares 0 and 24 h post-thaw cryopreservation outcomes. We first used a low DMSO concentration (2.5 wt %), rather than the more common 10 wt %, to allow any additive benefit from the polymers to be seen but to also avoid false-positives. This strategy has been used previously to screen polyampholytes.^32,51^ Stover *et al*. reported a macromolecular cryoprotectant which appeared to give excellent immediate post-thaw recovery, but the cells failed to survive in extended culture. However, addition of a small amount of DMSO in the freezing media did give improved cell recovery.^52^ Using a panel of PMSEM, varying in degree of polymerization from 50 to 200, there was no real benefit in cryopreservation compared to 2.5 wt % DMSO alone (Figure 5) across several technical and biological replicates. It was interesting to note the range of the reported results, with some outliers showing increases across replicates. Cryopreservation is impacted by a range of factors during the inubcation time, freezing and thawing processes, which may contribute to this. Regardless, the data set was clear that PMSEM did not enhance DMSO cryopreservation when using a standard slow cooling (−1 °C per minute to −80 °C) and rapid thawing rate. It is crucial to note that as so many factors occur in cryopreservation, one cannot rule out a set of conditions or a combination where a particular additive can help, and hence, there may be specific conditions where these materials could benefit. A possible explanation for the variable recovery data is that the sulfoxides are antioxidants. Tirelli has explored main-chain sulfoxide polymers in this context to reduce inflammation^53^ and antioxidants can improve post-thaw viability of cells,^54^ but the role of reactive oxygen species in A549 cells (to the best of our knowledge) has not been studied. Cryopreservation under identical conditions was attempted with PDMAOEM also, giving broadly similar results with no enhancement in post-thaw recovery (Figure S7, Supporting Information). PDMAOEM can be considered as a polymeric equivalent of TMAO (trimethyl amine oxide), which is a plant cryoprotectant. PDMAOEM, however, did give less variance than PMSEM, which supports the hypothesis that the variable oxidation states might be contributing to the cryopreservation outcomes: the PDMAOEM oxidation state cannot change under the conditions used.

**Figure 6:**
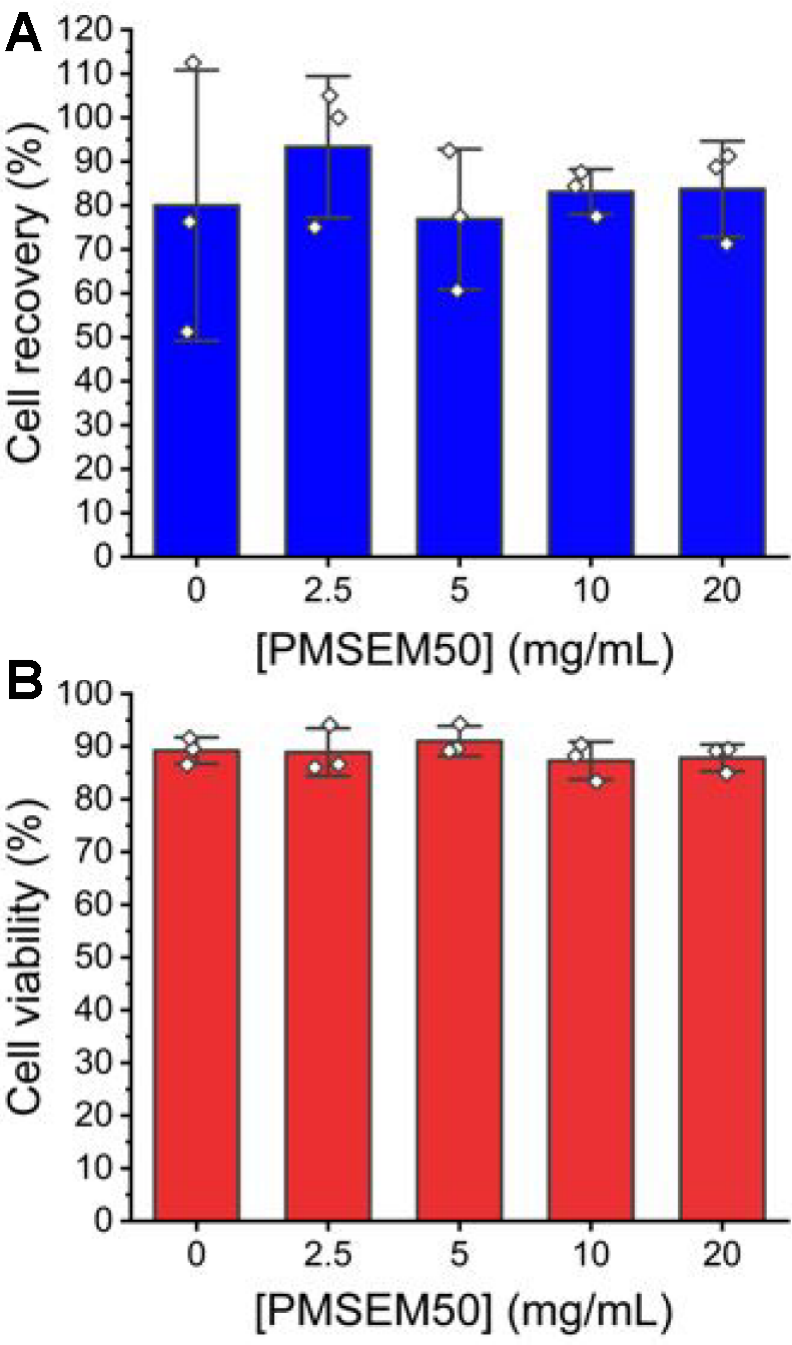
Cryopreservation of A549 cells (in suspension with 10 wt % DMSO and PMSEM_50_). Cell recovery (A) and viability (B) were determined 24 h post-thaw using the trypan blue exclusion test. Plots show three technical replicates each. Results are expressed as mean ± SD.

To allow comparison against more standard cryopreservation conditions, the experiments were repeated using 10 wt % DMSO and PMSEM. Figure 6 shows again that, under these conditions, there was no additive benefit of the polymer on either the total cell recovery or the viability of the recovered cells.

As an additional experiment to probe the cryopreservation process, dye-leakage assays from liposomes were undertaken, as polyampholytes have been shown to offer some protection to the cell membrane in these relatively simplistic models.^51,55^ At very high concentrations (200 mg mL^–1^), the PMSEMs showed some reduction in dye leakage (Figure S2) after freeze thaw (indicative of less damage), but the magnitude was less than that seen for polyampholytes, suggesting direct membrane protection was not occurring.

DMSO protects cells by replacing intracellular water, and hence, the cellular uptake of the PMSEM_100_ homopolymer was investigated. A fluorescently labeled polymer was synthesized by coupling *N*-(5-fluoresceinyl)maleimide, and A549 cell uptake was evaluated by fluorescence microscopy of the polymer with 2.5 wt % DMSO, to replicate the cryopreservation conditions. It was essential to conduct cell-uptake experiments under these conditions as DMSO is cell permeabilising, and hence, its inclusion avoids false-negatives compared with a media-only uptake experiment. Using fluorescence microscopy, no polymer-derived fluorescence was observed in the cells, suggesting limited uptake (Figure S10). As a more sensitive tool, flow cytometry was also employed. Using this, cell-associated fluorescence was observed in a dose-dependent manner, suggesting there was some cell uptake or association (Figure S11). However, flow cytometry cannot distinguish whether the observed fluorescence is due to surface-associated or internalized polymer. When combined with the microscopy results, this suggests insufficient polymer can enter the cell for a cryoprotective benefit when compared with DMSO, for example.

Taken together, this data set confirms that merely replicating a DMSO-like structure on a polymer side chain is not sufficient to provide a cryopreservation benefit. DMSO is cell permeable, reducing the likelihood of (fatal) intracellular ice formation, for example, and polymer immobilization will impact this. It may also suggest that the backbone chemistry of the polymers is crucial, as PEG-based polysulfoxides show a cryoprotective benefit but cannot be directly compared as identical freezing conditions were not used.^38^ This data is also interesting to compare to polyampholytes, which bear mixed cationic/anionic side chains and are very potent cryoprotectants but for which the mechanism of action is not clear.^15,31^ One key difference between polyampholytes and the polymers in this study is that the charges on polyampholytes occur on separate side chains, while for PMSEM and PDMAOEM, the charges occur on the same side chain, as is the case with polybetaines, which are also not cryoprotective.^52^

## Conclusions

Here we report the synthesis and characterization of 2-(methylthio)ethyl methacrylate and its subsequent conversion by postpolymerization oxidation to poly(2-(methylsulfinyl)ethyl methacrylate), PMSEM. The oxidation process introduces a sulfoxide unit that is intended to mimic the structure of DMSO, which is widely used in the cryopreservation of cells. These polymers were used to evaluate the role of the sulfoxide group on cryopreservation outcomes, to both understand if the DMSO-like functionality allows retention of its beneficial properties and to help guide the development of new macromolecular cryoprotectants by understanding the key motifs which give rise to protection.

Using ^1^H NMR spectroscopy, it was observed that overoxidation of the side chain generated copolymers with both sulfoxide and sulfone functionality. In initial cryopreservation and cytotoxicity studies, the sulphone-containing polymers showed significantly higher cytotoxicity, and hence, precision oxidation was required for all materials. PMSEM and a control *N*-oxide polymer were found to have no ice-recrystallization inhibition activity and to not significantly reduce the total ice fraction formed during freezing compared with a poly(ethylene glycol) control. The PMSEMs were found to be cytocompatible against A549 cells for short exposure periods (30 min), representative of the contact time in the liquid phase during cryopreservation. In longer-term culture (24 h), there was a dose-dependent decrease in cell viability. In cryopreservation assays using low (2.5 wt %) and standard (10 wt %) DMSO there was limited benefit by the addition of the PMSEM, with a substantial variance in post-thaw recoveries observed. This contrasts with a previous DMSO-like side chain polymer with a PEG background, which had moderate cryoprotective activity, suggesting the backbone hydrophilicity may be crucial. It also shows that the polarized sulfoxide group (with negative charge on oxygen and positive on sulfur) does not bring the same benefits on a polymer side chain as a polyampholyte with mixed cationic/anionic groups. Overall, we show that careful oxidation is essential in developing sulfoxide side-chain polymers for cryobiology and that simply introducing this unit does not guarantee a cryoprotective benefit, suggesting that the polymer backbone as well as the sulfoxide side chain need to be fine-tuned to gain a post-thaw benefit.

## Supporting Information Available

Full synthetic details and additional FTIR characterization of polymers; experimental details of cell based assays and additional cell viability and recovery data (PDF)

## Notes

Any additional research data supporting this publication can be found at http://wrap.warwick.ac.uk.
