# Supplementary material for: Synthesis of Poly(2-(methylsulfinyl)ethyl methacrylate) via Oxidation of Poly(2-(methylthio)ethyl methacrylate): Evaluation of the Sulfoxide Side Chain on Cryopreservation
Source: ACS Polym Au. 2022 Aug 5;2(6):449–57. doi: 10.1021/acspolymersau.2c00028 (PMC9756334; doi:10.1021/acspolymersau.2c00028)
Supplement: Supplementary file 1 — lg2c00028_si_001.pdf [file lg2c00028_si_001.pdf]

Supporting Information

"Synthesis of Poly(2-(methylsulfinyl)ethyl methacrylate) *via* Oxidation of Poly(2-(methylthio)ethyl methacrylate): Evaluation of the Sulfoxide Side Chain on Cryopreservation"

*Toru Ishibe,[a] Natalia Gonzalez-Martinez,[a] Panagiotis G. Georgiou,[a] Kathryn A. Murray,[a,b] and Matthew I. Gibson[a,b]**

[a] Department of Chemistry, University of Warwick, Gibbet Hill Road, CV4 7AL, Coventry, UK

[b] Division of Biomedical Sciences, Warwick Medical School, University of Warwick, Gibbet Hill Road, CV4 7AL, Coventry, UK

**Corresponding Author*: m.i.gibson@warwick.ac.uk (M.I.G.)

Experimental Section

Materials

All chemicals were used as supplied unless otherwise stated. 2-(Dimethylamino)ethyl methacrylate (98%), 2-(methylthio)ethyl methacrylate (96%), 2-cyano-2-propyl dodecyltrithiocarbonate (97%), 2,2'-azobis(2-methylpropionitrile) (AIBN, 98%), hydrogen peroxide (30% w/v aqueous solution), 5(6)-carboxyfluorescein (CF) (>95%), *N*-(5-fluoresceinyl)maleimide (≥90%), 1,2-dioleoyl-sn-glycero-3-phosphocholine (DOPC) in chloroform and all solvents were purchased from Sigma-Aldrich and used without further purification. All monomers were filtered through a plug of basic alumina to remove inhibitors prior to use. 2.5 g Triton X-100 was dissolved in 100 mL MiliQ water.

Physical and Analytical Methods

^{1}H NMR spectra were recorded on Bruker DPX-300 and DPX-400 spectrometers using deuterated solvents purchased from Sigma-Aldrich. Chemical shifts are reported relative to residual non-deuterated solvent. Chemical shifts of protons are reported as δ in parts per million (ppm) and are relative to solvent residual peak ($(CD_3)_2SO$, δ = 2.50 ppm).

Infrared data was recorded on a Bruker Vector 22 GI003097.

DSC was recorded on a Mettler-Toledo DSC1 with autosampler. Cooling to -50 °C from 10 °C (-0.5 °C/min), and then heating to 10 °C (+0.5 °C/min) under air conditions.

DLS Hydrodynamic diameters (Dh) and size distributions of particles were determined by dynamic light scattering (DLS) using a Malvern Zetasizer Nano ZS with a 4 mW He-Ne 633 nm laser module operating at 25 ^{o}C. Measurements were carried out at an angle of 173° (back scattering), and results were analysed using Malvern DTS 7.03 software. All determinations

were repeated 5 times with at least 10 measurements recorded for each run. D_h values were calculated using the Stokes-Einstein equation where particles are assumed to be spherical.

Size exclusion chromatography (SEC) in DMF. Size exclusion chromatography (SEC) analysis was performed on an Agilent Infinity II MDS instrument equipped with differential refractive index (DRI), viscometry (VS), dual angle light scatter (LS) and variable wavelength UV detectors. The system was equipped with 2 x PLgel Mixed D columns (300 x 7.5 mm) and a PLgel 5 µm guard column. The mobile phase used was DMF (HPLC grade) containing 5 mM NH_4BF_4 at 50 °C at flow rate of 1.0 mL.min^{-1}. Poly(methyl methacrylate) (PMMA) standards (Agilent EasyVials) were used for calibration between 955,000 – 550 g.mol^{-1}. Analyte samples were filtered through a nylon membrane with 0.22 μm pore size before injection. Number average molecular weights (M_n), weight average molecular weights (M_w) and dispersities ($Đ_M = M_w/M_n$) were determined by conventional calibration and universal calibration using Agilent GPC/SEC software.

Size exclusion chromatography (SEC) in THF. Size exclusion chromatography (SEC) analysis was performed on an Agilent Infinity II MDS instrument equipped with differential refractive index (DRI), viscometry (VS), dual angle light scatter (LS) and multiple wavelength UV detectors. The system was equipped with 2 x PLgel Mixed C columns (300 x 7.5 mm) and a PLgel 5 µm guard column. The eluent was THF containing 2 % triethylamine (TEA) and 0.01 % BHT (butylated hydroxytoluene) additives. Samples were run at 1 mL.min^{-1} at 30 °C. Polystyrene (PS) standards (Agilent EasyVials) were used for calibration. Analyte samples were filtered through a GVHP membrane with 0.22 μm pore size before injection. Number average molecular weights (M_n), weight average molecular weights (M_w) and dispersities ($Đ_M = M_w/M_n$) were determined by conventional calibration and universal calibration using Agilent GPC/SEC software.

Ice Recrystallisation Inhibition Assay

A 10 µL sample of polymer dissolved in PBS buffer (pH 7.4) is dropped 1.40 m onto a glass microscope coverslip, which is on top of an aluminium plate cooled to −78 °C using dry ice. The droplet freezes instantly upon impact with the plate, spreading out and forming a thin wafer of ice. This wafer is then placed on a liquid nitrogen cooled cryostage held at −8 °C. The wafer is then left to anneal for 30 min at −8 °C. The number of crystals in the image is counted, again using ImageJ, and the area of the field of view divided by this number of crystals to give the average crystal size per wafer, and reported as a % of area compared to PBS control.

CF Leakage from Liposome Assay

DOPC in chloroform (1.0 mL, 25 mg/mL) was evaporated to produce a thin lipid film. The film was dried for an hour under a vacuum. The resulting lipid film was suspended in a 0.1 M CF/PBS solution (1.0 mL) to be sonicated for 30 minutes. The process of freezing and thawing the aqueous solution was repeated five times. The unilamellar vesicles were obtained using a mini-extruder set (Avanti Polar Lipids) and membranes with a 0.1 µm pore size. The excess of CF dye was removed by passing the liposome though a PD-10 column (GE Healthcare). The liposome size was measured by Dynamic light scattering (DLS) (Figure S1). The resultant liposome solution (0.15 mL) and the polymer solution (0.15 mL) were mixed and separated to three Eppendorf tubes, 0.1 mL each. One of these tubes was frozen by liquid nitrogen for 10 min and then thawed at 37 ºC for 5 min. For complete dye leakage (100% leakage), Triton X-100 (3 mg) was added to other tube. The third tube was used for the measurement of the initial fluorescence. CF fluorescence was measured with BioTek Synergy HT Microplate Reader using an excitation wavelength of 485 nm and a detection wavelength of 528 nm. Fluorescence was quenched when the vesicles were protected after the freeze-thaw process and increase in

fluorescence intensity represented membrane damage due to the release of CF into the PBS solution.

Synthesis of Poly(2-(methylsulfinyl)ethyl methacrylate) (PMSEM)

As a representative example, 2-(methylthio)ethyl methacrylate (2.00 g, 12.5 mmol, 50 eq), 2-cyano-2-propyl dodecyl trithiocarbonate (0.086 g, 0.25 mmol, 1 eq), 2,2'-azobis(2-methylpropionitrile) (0.004 g, 0.024 mmol, 0.1 eq), THF (6 mL) as solvent, and DMF (70 μL) as an NMR standard were added to round-bottom flask. A stirrer bar was added, the flask sealed with a suba seal, and the solution bubbled with nitrogen for 20 minutes. After this time, a small sample was removed to determine conversion, and the flask was placed into an oil bath set to 60 ºC. After 24 hours, another sample was taken for conversion at ambient temperature. Hydrogen peroxide (30% w/v aqueous solution, 2.8 g, 24.7 mmol, 100 eq) was added to the resulting polymer solution, and the flask was placed into an oil bath set to 40 ºC. After 18 hours, the polymer was precipitated into acetone, dissolved in water, and dialysed (1000 Da MWCO) for 48 hours (7 water changes), followed by lyophilisation. The resulting polymer was analysed by ^{1}H NMR, FTIR and SEC. Representative characterisation data for $PMSEM_{50}$: ^{1}H NMR (400 MHz, DMSO): δ = 0.82 – 0.99 ($CH_2C(\mathit{CH_3})$, br), 1.80 – 1.87 ($\mathit{CH_2}C(CH_3)$, br), 2.65 ($\mathit{CH_3}S(O)CH_2CH_2O$, br), 3.00 – 3.16 ($CH_3S(O)\mathit{CH_2C}H_2O$, br), 4.19 – 4.35 ($CH_3S(O)CH_2\mathit{CH_2}O$, br). FTIR: S=O 1028 cm^{-1}, C-O 1144 cm^{-1}, C=O 1722 cm^{-1}. $M_n^{SEC\ RI}$ (DMF) = 12,000 Da, $Đ_M$ = 1.19.

Synthesis of Poly(2-(dimethylamineoxide)ethyl methacrylate)) (PDMAOEM)

As a representative example, 2-(dimethylamino)ethyl methacrylate (2.98 g, 19.0 mmol, 50 eq), 2-cyano-2-propyl dodecyl trithiocarbonate (0.131 g, 0.38 mmol, 1 eq), 2,2'-azobis(2-methylpropionitrile) (0.006 g, 0.037 mmol, 0.1 eq), THF (9 mL) as solvent, and DMF (100 μL)

as an NMR standard were added to round-bottom flask. A stirrer bar was added, the flask sealed with a suba seal, and the solution bubbled with nitrogen for 20 minutes. After this time, a small sample was removed to determine conversion, and the flask was placed into an oil bath set to 60 ºC. After 24 hours, the polymer solution including poly(2-(dimethylamino)ethyl methacrylate) (PDMAEM) was taken for the analysis of ^{1}H NMR, FTIR and SEC at ambient temperature. Hydrogen peroxide (30% w/v aqueous solution, 4.3 g, 37.9 mmol, 100 eq) was added to the resulting polymer solution, and the flask was placed into an oil bath set to 40 ºC. After 18 hours, the polymer was precipitated into acetone, dissolved in water, and dialysed (1000 MWCO) for 48 hours (7 water changes), followed by lyophilisation. The resulting polymer was analysed by ^{1}H NMR, FTIR. Representative characterisation data for $PDMAEM_{50}$: ^{1}H NMR (400 MHz, $CDCl_3$): δ = 0.87 – 1.08 ($CH_2C(\mathit{CH_3})$, br), 1.81 – 1.92 ($\mathit{CH_2}C(CH_3)$, br), 2.28 ($(\mathit{CH_3})_2NCH_2CH_2O$, br), 2.53 – 2.58 ($(CH_3)_2N\mathit{CH_2}CH_2O$, br), 4.03 – 4.09 ($(CH_3)_2NCH_2\mathit{CH_2}O$, br). FTIR: C-O 1146 cm^{-1}, C-N 1260 cm^{-1}, C=O 1720 cm^{-1}. $M_n^{SEC\ RI}$ (THF) = 4000 Da, $Đ_M$ = 1.38.

$PDMAOEM_{50}$: ^{1}H NMR (400 MHz, D_2O): δ = 0.83 – 1.10 ($CH_2C(\mathit{CH_3})$, br), 1.82 – 2.00 ($C\mathit{H_2}C(CH_3)$, br), 3.23 ($(C\mathit{H_3})_2N(O)CH_2CH_2O$, br), 3.60 – 3.69 ($(CH_3)_2N(O)C\mathit{H_2}CH_2O$, br), 4.36 – 4.47 ($(CH_3)_2N(O)CH_2C\mathit{H_2}O$, br). FTIR: C-O 1146 cm^{-1}, C-N 1233 cm^{-1}, N-O 1450 cm^{-1}, C=O 1720 cm^{-1}.

Conjugation of Poly(2-(methylsulfinyl)ethyl methacrylate) ($PMSEM_{100}$) with *N*-(5-Fluoresceinyl)Maleimide

Poly(2-(methylsulfinyl)ethyl methacrylate) ($PMSEM_{100}$) (0.3 g, 0.017 mmol, 1 eq) and *N*-(5-fluoresceinyl)maleimide (0.002 g, 0.034 mmol, 2 eq) were dissolved in a round bottom flask using 5 mL of anhydrous DCM. A stirrer bar was added, the flask sealed with a suba seal, and the solution bubbled with nitrogen overnight. Resulted reaction solution was precipitated in

acetone and then redissolved in water and dialysed for 3 days (MWCO = 500 Da) (7 water changes) followed by lyophilisation resulting to the formation of an orange polymer powder. To confirm successful conjugation of fluorescent maleimide, UV-Vis analysis was performed on polymer solution before and after conjugation (Figure S9).

Cell culture

Adenocarcinomic human alveolar basal epithelial (A549) cells were cultured in 175 cm^2 cell culture flasks (Corning). The cell culture media consisted of Ham's F12-K media (Gibco) supplemented with 10% foetal bovine serum (FBS) (Sigma-Aldrich) and 1% antibiotic-antimycotic solution containing 10,000 units mL^{-1} of penicillin, 10 mg mL^{-1} streptomycin, and 25 μg mL^{-1} amphotericin B (Sigma-Aldrich). A549 cells were maintained at 37 °C and 5% CO_2 in an incubator and passaged when they reached 80% confluency, normally every 3-4 days. To passage cells, these were dissociated from the flask by treating them with 0.25% Trypsin in EDTA (Gibco).

Cell suspension cryopreservation

After dissociation, A549 cells were centrifuged at 2000 rpm for 5 minutes and resuspended using freezing media, which was supplemented with 10% FBS only. An aliquot of cells was taken, and this aliquot was diluted 1:1 with 0.2% trypan blue. The number of cells with intact membranes (unstained cells) were counted using a haemocytometer (Sigma Aldrich) and the cell density was adjusted to 8 x 10^5 cells mL^{-1}**.** This cell counting method is referred to in text as the trypan blue exclusion test. The polymers were dissolved in freezing media supplemented with DMSO at twice the final concentration (for example, 40 mg mL^{-1} in 5% DMSO for a final concentration of 20 mg mL^{-1} in 2.5% DMSO) and sterilised using a 0.2 µm filter (Sartorius). Cells were then diluted 1:1 in the freezing media supplemented with DMSO and the polymers, and a total of 4 x 10^5 cells were frozen per cryovial in 1 mL. Experimental conditions were

assessed in triplicates. Cryovials (Sigma-Aldrich) were then placed in a CoolCell LX vial freezing container (Corning) and into a -80 °C freezer to cool at a rate of -1 °C per minute. After 24 hours, cryovials were thawed in a water bath at 37°C for 2-3 minutes, until no ice crystals were visible. The cell suspension from each cryovial was diluted in 9mL of warm cell culture media, centrifuged at 2000 rpm for 5 minutes, resuspended using 400 µL of cell culture media and transferred into a 24 well plate (Greiner). A549 cells were incubated at 37 °C and 5% CO_2. After 24 hours, cell counts were performed using the trypan blue exclusion test as previously described and cell recovery (%) was calculated by dividing the number of unstained cells obtained post-thaw by the initial number of cells frozen. To assess cell viability 24 hours post-thaw, cell culture media from the supernatant was collected before cellular dissociation. Non-attached cells were considered as dead as they failed to attach to the plate. Cell viability was therefore calculated by dividing the number of unstained cells post-thaw by the number of stained plus non-attached cells.

Polymer cytotoxicity assay

Polymer cytotoxicity was tested by measuring the metabolic reduction of resazurin to resorufin as an indicator of cell viability after either a 30-minute or 24-hour incubation period. A549 cells were seeded at 1 x 10^4 cells per well in 100 µL of cell culture media in 96-well plates. After 24 hours, the cell culture media was replaced with 100 µL of media supplemented with decreasing polymer concentrations from 40 to 1.25 mg mL^{-1}. The cells were then incubated with the polymer solutions for either 30 minutes or 24 hours at 37 °C and 5% CO_2. One tablet of resazurin sodium salt (Scientific Laboratory Supplies) was diluted 1 in 10 in cell culture media. After 24 hours, cell culture media was discarded and 100 µL of the resazurin sodium salt mixture was added to all wells. Cells were incubated for 1-4 hours at 37°C and 5% CO_2 and the absorbance was measured hourly using the Synergy HTX Multi-Mode Reader (BioTek) at 570 and 600 nm. Measurements were performed until the control cells reached

approximately 80% resazurin reduction. To calculate cell viability, the treated cells were compared to the untreated control cells and the result was expressed as a percentage.

Cellular uptake studies using fluorescent microscopy

A549 cells were seeded in a 24 well plate at 5 x 10^4 cells per well in 500µL of cell culture media. After 24 hours, cell culture media was discarded and cells were incubated for 30 minutes at room temperature with 20 mg mL^{-1} of the fluorescently-labelled PMSEM100 in cell culture media containing 2.5% DMSO. Wells were then washed 3 times with Dulbecco's Phosphate Buffered Saline (DPBS) and cells were stained with 100 µg mL^{-1} Hoechst 33342 nuclear stain (Thermo Scientific) in DPBS for 5 minutes at room temperature. Wells were washed 3 times in DPBS before imaging. Negative control wells were incubated with 2.5% DMSO for 30 minutes and stained with Hoechst 33342 only. Positive control wells were incubated with 2.5% DMSO for 30 minutes, Hoechst 33342 and 1 µg mL^{-1} Rhodamine 123 (Vector Laboratories) for 20 minutes at room temperature to stain the mitochondria of live cells. This dye was chosen as it fluoresces at the same channel as the fluorescently labelled polymer (green/FITC channel). Imaging was completed with a 10x objective lens, 380-405 nm and 451-488nm excitation filters and 416-452, 502-532 emission filters using an Olympus IX83 microscope. Images were analysed and overlayed using the Olympus ScanR analysis software.

Cellular uptake studies using flow cytometry

A549 cells were seeded in a 24 well plate at 5 x 10^4 cells per well in 500µL of cell culture media. After 24 hours, media was discarded and cells were incubated for 30 minutes at room temperature with different concentrations (20 mg mL^{-1} to 1.25 mg mL^{-1}) of the fluorescently labelled PMSEM100 in cell culture media containing 2.5% DMSO. Then, cells were washed 3 times in DPBS. Negative controls were prepared by incubating cells in cell culture media containing 2.5% DMSO for 30 minutes. Cells were dissociated from the plate by treating them

with 0.25% trypsin in EDTA and subsequently, trypsin was inactivated by a 1:1 dilution with cell culture media. Flow cytometry of the cell suspension was then performed in a BD Accuri C6 flow cytometer. Fluorescence was measured using the 488 nm excitation laser and 530/30 nm emission filter and 20,000 events were acquired per sample. FlowJo was used to analyse and plot flow cytometry data.

Additional Data

Additional control experiments using liposomes were undertaken as simplistic cell models. Figure S1 shows DOPC liposomes with and without addition of PMSEM. This showed that the polymers did not disrupt nor aggregate the liposomes, which was essential to allow further freeze/thaw testing (see Figure S2).

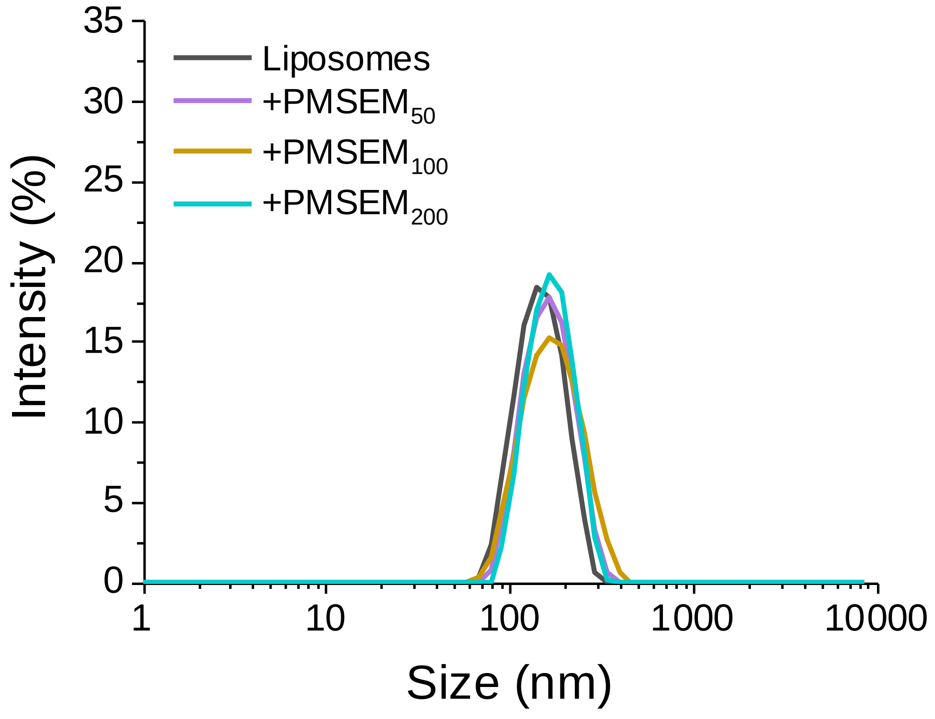

Figure S1. Dynamic light scattering analysis of DOPC (dioleoylphosphatidylcholine) with addition of 10 mg.mL^{-1} of PMSEM polymers.

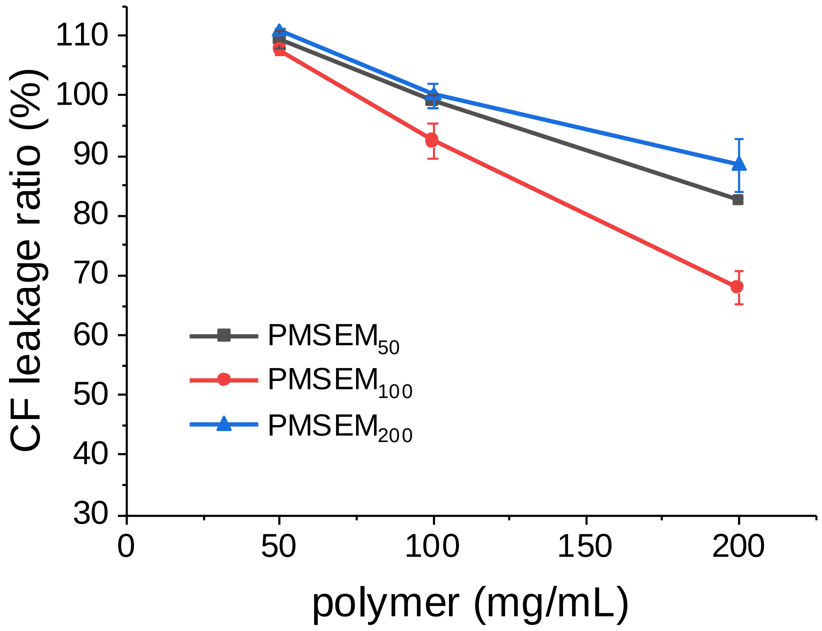

Figure S2. Leakage of carboxyfluorescein from DOPC (dioleoylphosphatidylcholine) liposomes after freeze (in liq. N_2)/thaw (37 °C). Results are reported relative to a 100 % lysis control using Triton X. Error is the standard deviation from 3 replicates.

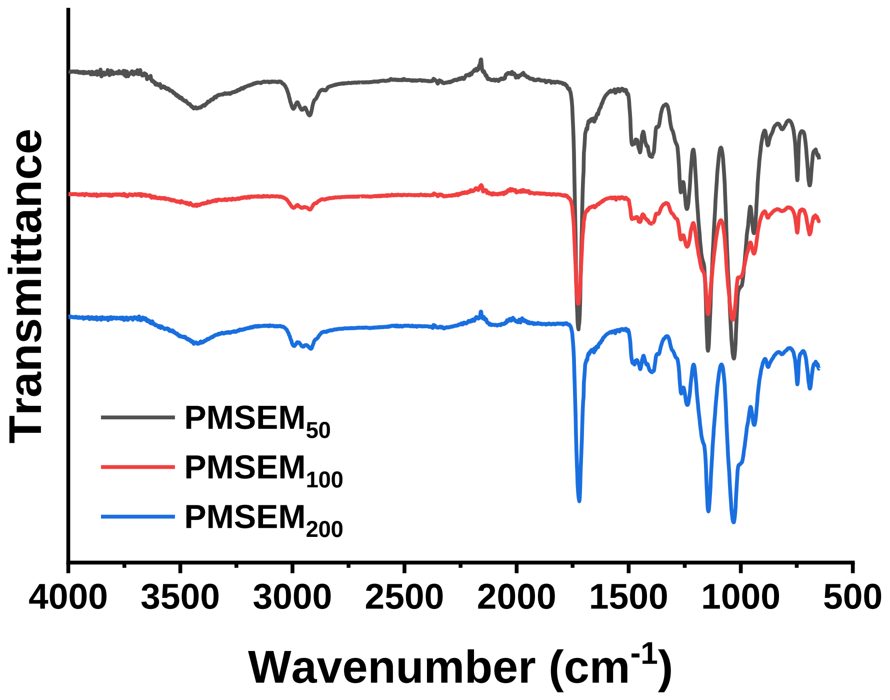

Figure S3. Infrared spectra of PMSEM polymers

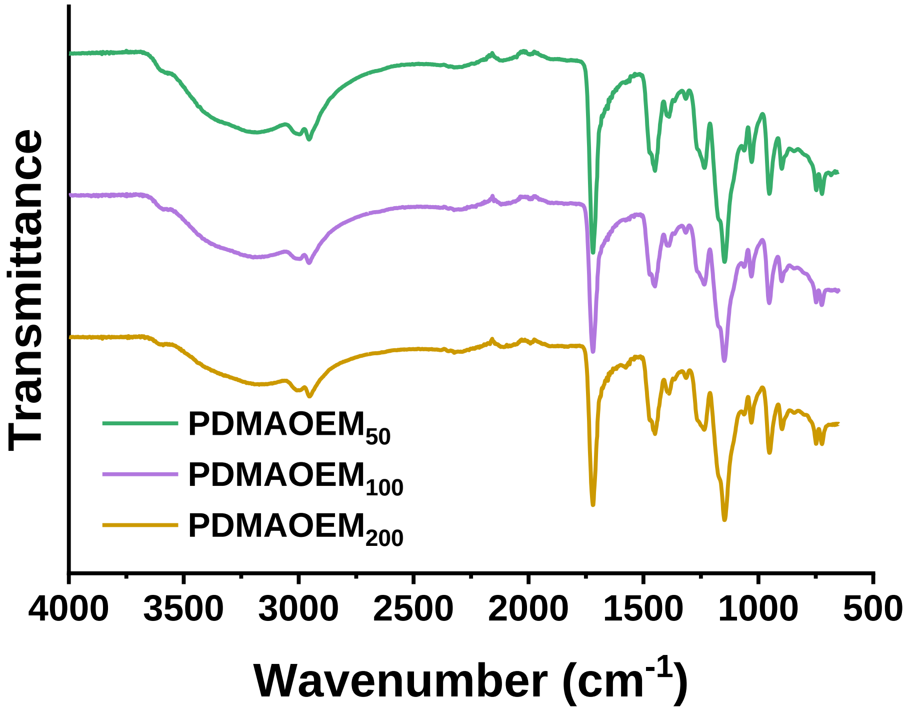

Figure S4. Infrared spectra of PDMAOEM polymers

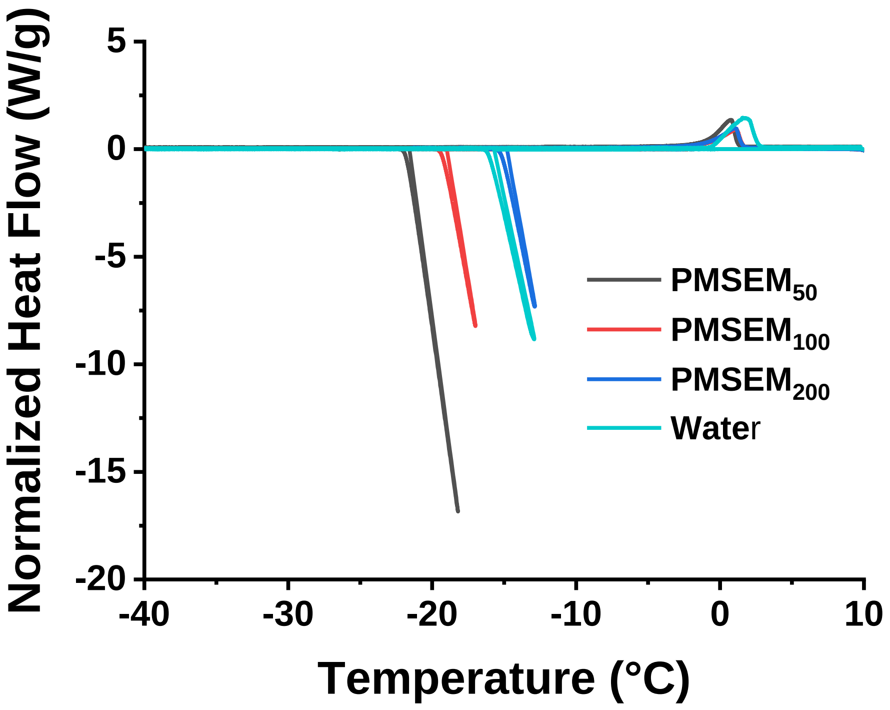

Figure S5. Differential scanning calorimetry of PMSEM polymers in water.

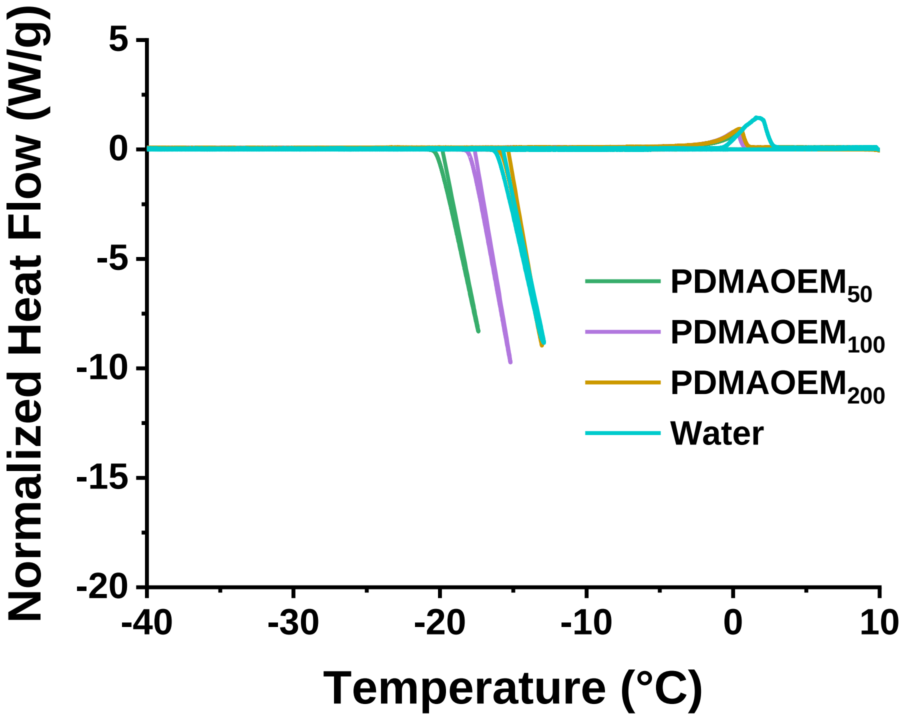

Figure S6. Differential scanning calorimetry of PDMAOEM polymers in water.

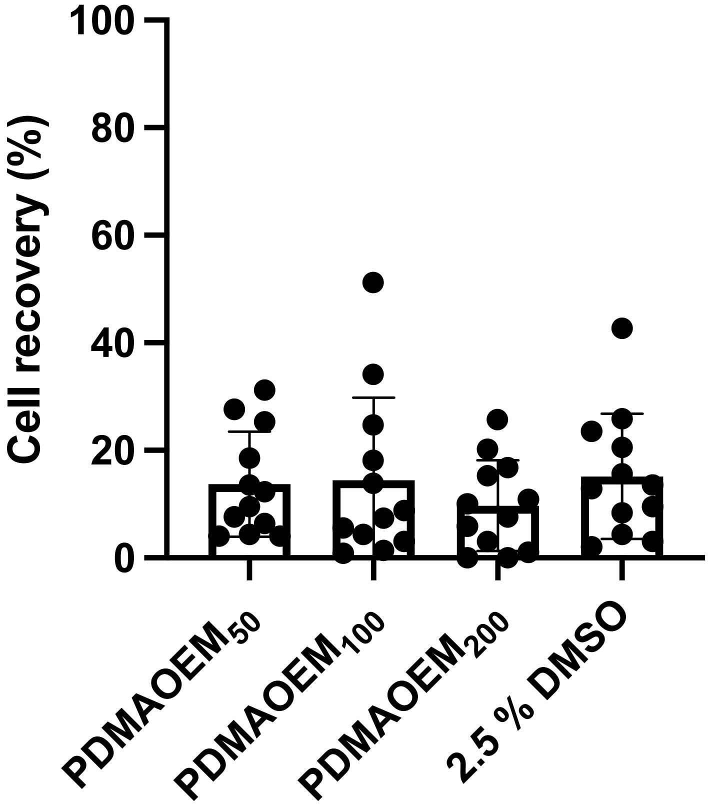

Figure S7. Cryopreservation of A549 cells (in suspension) as a function of *N*-oxide polymer molecular weight. Cell recovery was determined 24 hours post-thaw using trypan blue exclusion test. Results expressed as mean ± SD. Plots show 3 biological repeats with 3 technical replicates each.

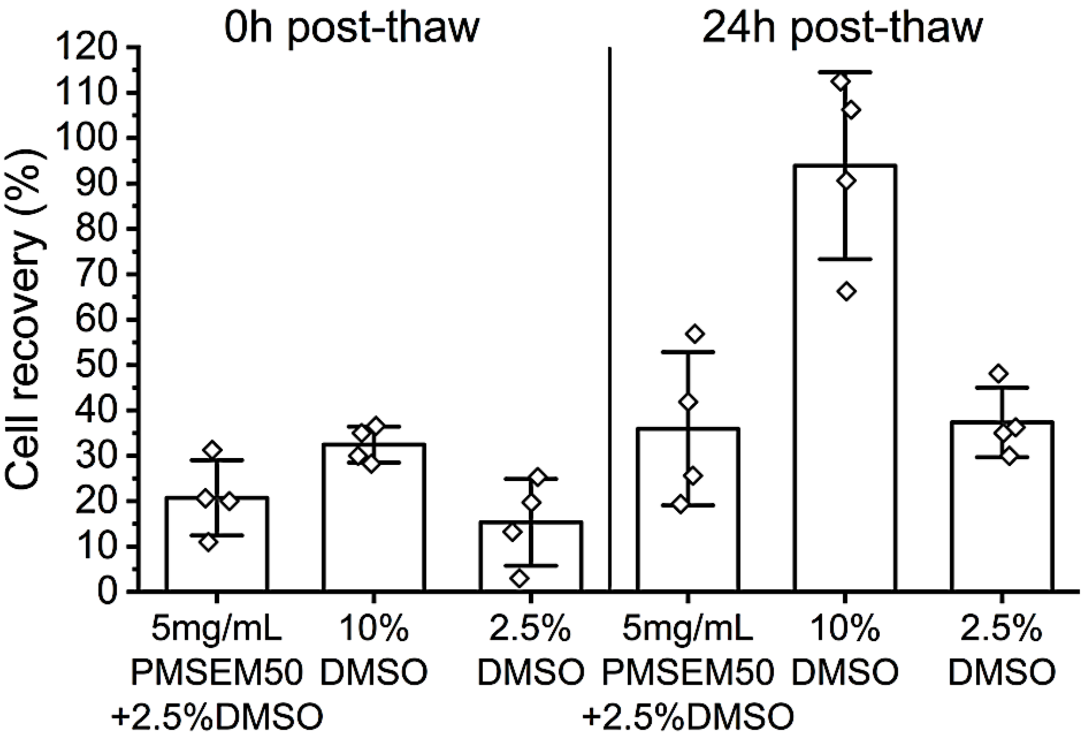

Figure S8. Post-thaw A549 cell recovery following suspension cryopreservation with the indicated additives. Calculated using trypan blue exclusion test. Plots show 4 technical replicates each. Results expressed as mean ± SD.

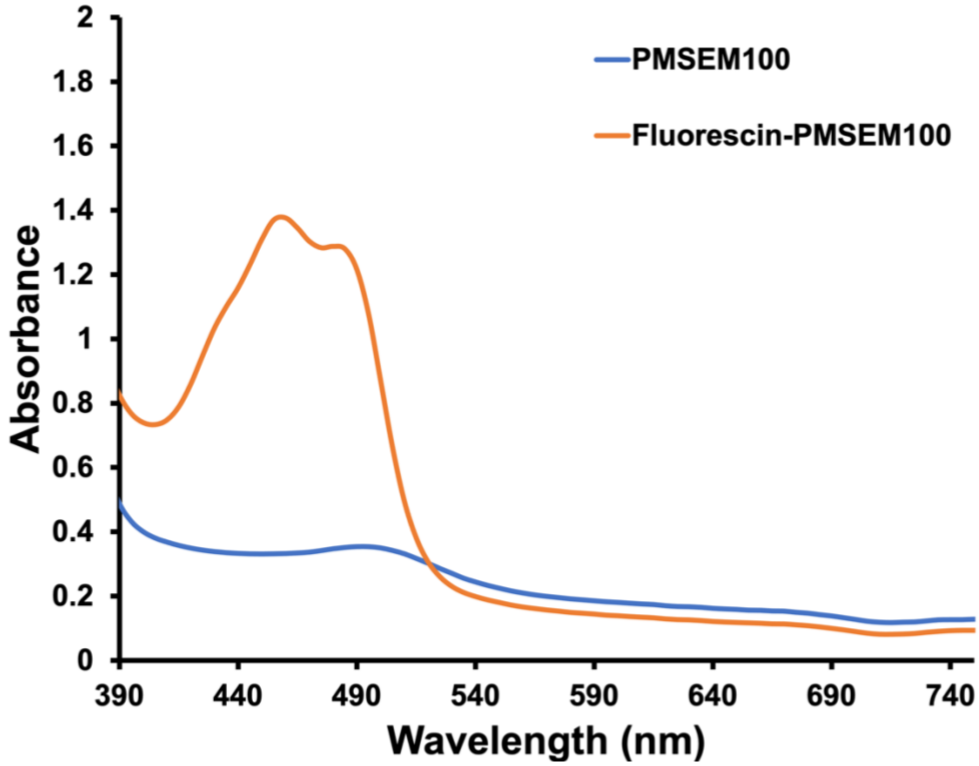

Figure S9. UV-Visible spectra for 2 mg.ml^{-1} of $PMSEM_{100}$ and fluorescin-conjugated-$PMSEM_{100}$.

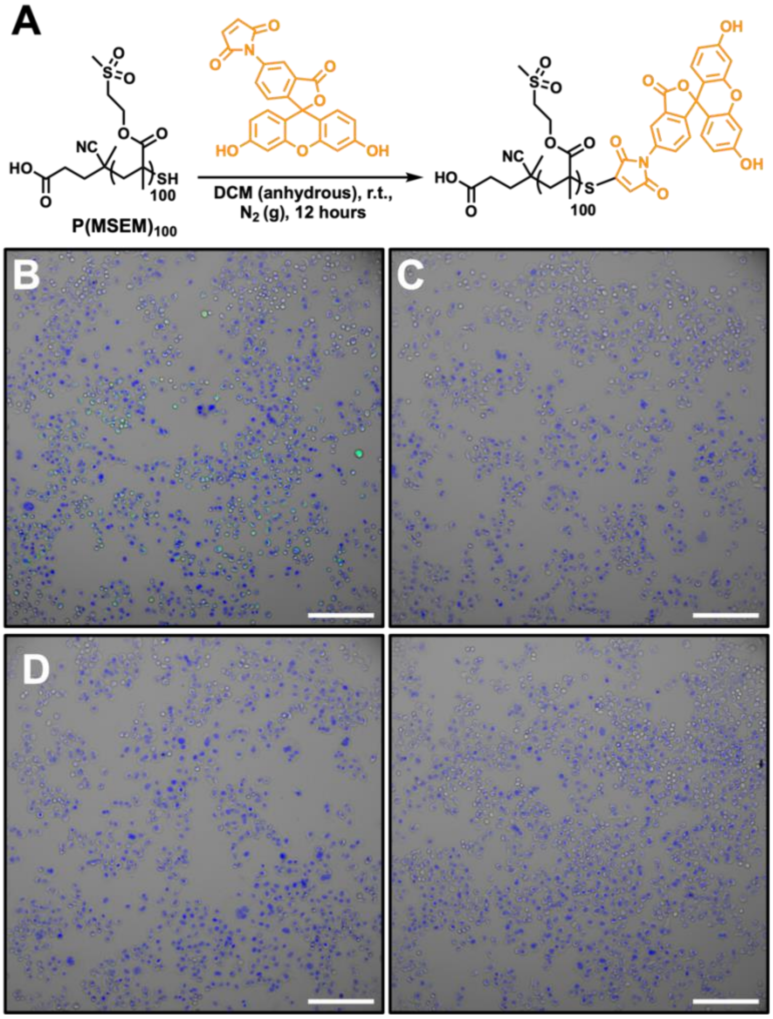

Figure S10. Fluorescence microscopy of cell uptake. A. Conjugation of $PMSEM_{100}$ with *N*-(5-fluoresceinyl)maleimide. B. shows positive control of cells stained with Rhodamine 123 mitochondrial stain (green) and Hoechst nuclear stain (blue). C shows negative control of cells stained with Hoechst nuclear dye only. B and C were incubated with 2.5% DMSO for 30 minutes at room temperature. D shows cells incubated with 20 mg/mL of fluorescently labelled $PMSEM_{100}$ in 2.5% DMSO for 30 minutes at room temperature and stained with Hoechst nuclear dye only. Scale bar represents 200 µm. Imaging was completed using a 10x objective lens. Fluorescence was detected using 380-405 nm and 451-488nm excitation filters and 416-452, 502-532 emission filters optimised for the detection of Hoechst (DAPI channel) and fluorescently labelled polymer (FITC channel) using an Olympus IX83 microscope.

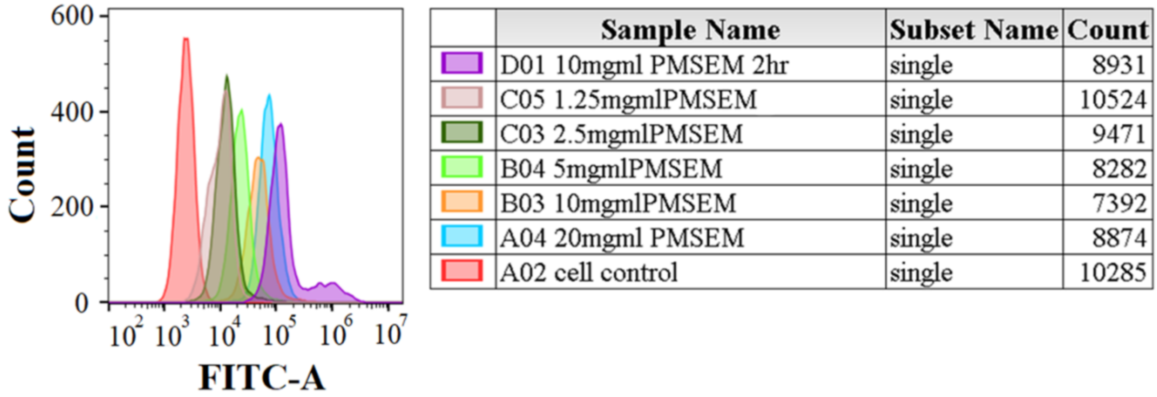

Figure S11. Relative cell-associated fluorescence measured by flow cytometry. Cells were incubated for 30 minutes at room temperature with varying concentrations (20 mg mL^{-1} to 1.25 mg mL^{-1}) of fluorescently labelled $PMSEM_{100}$ in cell culture media containing 2.5% DMSO. Cell control was incubated for 30 minutes with media containing 2.5% DMSO. Fluorescence was detected using the 488 nm excitation laser and 530/30 nm emission filter using a BD Accuri C6 Plus flow cytometer.
